# The Mediating Role of the Self-Concept Between the Relationship of the Body Satisfaction and the Intention to Be Physically Active in Primary School Students

**DOI:** 10.3389/fpubh.2020.00113

**Published:** 2020-05-08

**Authors:** Pedro Antonio Sánchez-Miguel, Patxi León-Guereño, Miguel Angel Tapia-Serrano, David Hortigüela-Alcalá, Miguel A. López-Gajardo, Mikel Vaquero-Solís

**Affiliations:** ^1^Faculty of Teaching Training, University of Extremadura, Cáceres, Spain; ^2^Faculty of Psychology and Education, University of Deusto, Donostia-San Sebastian, Spain; ^3^Department of Specific Didactics, Faculty of Education, University of Burgos, Burgos, Spain; ^4^Faculty of Sports Sciences, University of Extremadura, Cáceres, Spain

**Keywords:** body image, physical fitness, physical condition, youths, primary school

## Abstract

The aim was to analyze the extent to which anthropometric values, in line with body image and physical ability, predict physical self-concept, and the latter, in turn, predicts the practice and intention to pursue physical activity. A total of 302 participants, 150 males and 152 females were recruited from different primary schools in Extremadura (Spain). The age of the participants ranges from 10 to 13 years old (*M* = 11.74; *SD* = 0.86). The indirect effects of the model showed significant relationship between physical condition (*p* = 0.001) and PA levels, according to the perception of self-concept [β = 0.231, 95% BcCI = (0.055, 0.212)]. However, anthropometric variables proved not to be related to any significant extent (*p* < 0.05). The second level covered the indirect effects between the intention to be physically active and self-concept, which showed a significant relationship between the perception of self-concept (*p* = 0.000) and the intention to be physically active. Last, the third level showed significant relationships between physical condition (*p* = 0.001) and the intention to pursue physical activity. The present investigation concluded that physical condition, anthropometric variables, and body image predict the perception of physical self-concept in adolescents. Finally, this article highlights the importance of body image perception, anthropometric values, and physical condition in the intention of being physically active. In addition, it highlights the mediating role of physical self-concept to develop physical activity.

## Introduction

Adolescence is characterized by being a stage in which major biological and psychological changes take place that define what a person will be like in adulthood (1). Studies show a decline in physical activity (PA) during this stage (2, 3), and this is a cause for concern given the physical and psychosocial benefits gained from doing PA (4), such as improvements in cardiovascular fitness, anthropometric values, bone structure, reduction in symptoms associated with depression and anxiety, and an improvement in psychological well-being. Along these lines, the adolescent stage is characterized by being a critical period in the development of the body image and one's perception of physical self-concept, whereby poor development of these constructs may give rise to problems linked to depression, anxiety, eating disorders, and imbalances in the perception of the body image and self-concept (5–7). In this respect, Casas et al. (8) stress the importance of detecting any factors that may contribute to an improvement in mental health in young people, drawing attention to the influence of body weight. With this in mind, Fernández-Bustos et al. (9) consider the body mass index (BMI) to be a major predictor of body image and self-esteem as well as body dissatisfaction in both men and women (10).

In keeping with the above, body image is a key element in the shaping of one's self-concept (11), and in this sense, body image reflects how individuals think, are viewed and act (12). Añez et al. (13) show its relationship with PA and draw attention to the fact that a poor body image may act as a barrier to pursuing physical activity. Moreover Neumark-Sztainer et al. (14), explain that adolescents with low body satisfaction are less likely to commit themselves to physical activity, instead of being more likely to spend their time indulging in sedentary activities such as watching TV, playing video games, or using the phone, etc. Likewise, attention is drawn to the moderating role of gender in associating body image with PA, by showing a positive association between the two in the case of the male gender (15). Conversely, there is no clear evidence of physical exercise in body image in the case of the female gender (16).

Additionally, body image is a factor that has a great impact on psychological well-being, which determines how self-concept is shaped, in particular, during adolescence (11). Numerous studies refer to a positive relationship between body satisfaction and self-concept (17, 18). Basing ourselves on the hierarchical and multidimensional model of general self-concept (19), physical self-concept (20) comprises the sum of specific domains: perceived competence, physical condition, physical appeal, and strength, while Sonstroem et al. (21) go on to add self-esteem. Previous studies have tended to relate physical self-concept to physical activity (9, 22, 23), the intention to pursue physical activity, social support (24), body image (25), physical condition (26), and anthropometric values such as BMI (27).

In terms of theoretical models that attempt to provide an explanation about the determining factors that the pursuit of physical activity entails, Fernández-Bustos et al. (9) explain that both physical activity and BMI predict body image, physical self-concept, and general self-concept. In this regard, Garn et al. (28) and Zamani Sani et al. (29) draw attention, respectively, to reciprocity and direct and indirect links to physical and psychological mechanisms. Similarly, Jekauc et al. (30) focus on the importance of motor skills in predicting self-concept and, ultimately, PA, while Moreno et al. (24), Fernandez-Rio et al. (31), and Grao Cruces et al. (32) point out that it is physical self-concept that predicts the intention to be physically active. Last, Li et al. (33) propose a model in which social support (parents and peers) predicts physical self-concept, and the latter, in turn, predicts physical activity.

For all the aforementioned reasons, the present research tests a model based on four levels, in which anthropometric values, in line with body image and physical ability (level 1), predict self-concept (level 2), which will, in turn, predict physical activity (level 3), and this last-mentioned will predict the intention to pursue it (level 4). Some authors differ in how they test their models, drawing attention to distinct variables when predicting self-concept and the relationship this has to the previously mentioned variables.

Hence, this work constitutes an attempt to provide a new approach that may offer an explanation about the physical and psychological mechanisms associated with the practice of, and intention to, pursue PA. For this reason, the aim of this research is to analyze the extent to which anthropometric values, in line with body image and physical ability, predict physical self-concept, and the latter, in turn, predicts the practice and intention to pursue PA. To this end, anthropometric values, physical condition, and body image are thought to provide a positive prediction of the perception of self-concept, physical activity, and the intention to pursue the latter, while the perception of self-concept is likewise thought to act as mediator between anthropometric values, physical condition, body image, and the intention to pursue physical activity.

## Materials and Methods

### Participants

A total number of 302 Spanish students from eight different primary schools in Extremadura, Spain, agreed to participate on a voluntary basis: males (*n* = 150) and females (*n* = 152), ranging in age from 10 to 13 years old (*M* = 11.74; *SD* = 0.86). All data was collected during normal school hours. The sample was selected according to the relevant research, respecting accessibility and involvement on the part of the schools. Moreover, taking into account that the highest levels of physical inactivity were evidenced in adolescents (7), we would like to cover school ages in order to test the relationships between the different variables. Data was collected from February 2018 to May 2018 in Cáceres. The sample was selected through hierarchical cluster sampling, considering the distance of the schools, whether the teaching staff was available, and the time required for the researcher to travel.

### Instruments

#### Anthropometric Variables

##### Body Mass Index

The body mass index was established by obtaining weight and height, and by applying the following formula: weight (kg)/height (m)^2^. Height and weight were established using the SECA 884 stadiometer model.

##### Waist-to-Height Ratio (WHtR)

The waist height perimeter was used, in which previous research has related to cardiometabolic markers (34). *WHtR* was calculated by dividing waist circumference (in cm) by height (in cm) (35).

#### Physical Condition Variables

##### Aerobic capacity

This was assessed using the 6-min walk test (6MWT), which was standardized by the American Thorax Society (36). A single 6MWT was undertaken along a flat, straight corridor over a hard surface, and the stimulus was used during the test. The children were given instructions to stop if they felt upset or uncomfortable. The maximum recorded distance (6MWD) was determined at the end of the test in order to assess the children's cardiorespiratory level. With a view to establishing comparisons, raw data was used owing to a possible bias in obtaining an equation in order to estimate the mean peak VO2 from the mean 6MWD in the children who took part (37).

#### Psychosocial Variables

##### Body image

**Figure 1:**
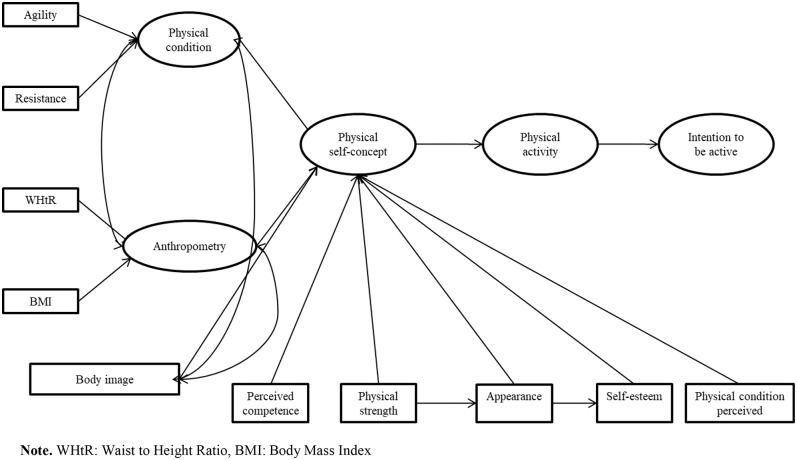
Hypothesized model. WHtR, waist-to-height ratio; BMI, body mass index.

The Stunkard Figure Rating Scale (Figure 1) was used to assess self and ideal body sizes. The Stunkard Scale consists of nine silhouette figures that increase in size from very thin (a value of 1) to very obese (a value of 9) (38). Self-body size refers to the number of the figure selected by participants in response to the prompt “Choose the figure that reflects how you think you look.” Ideal body size refers to the number of the figure chosen in response to the prompt “Choose your ideal figure.” For self-body size and ideal body size, dummy variables were created for the underweight, normal weight, overweight, and obese body size categories. This scale evidenced good validity and reliability (39), and body size satisfaction was subsequently calculated. The ensuing value is defined as the difference between one's perceived self-body size and perceived ideal body size. A body size satisfaction value was created for each participant by subtracting the number of the figure indicated as being the ideal body size from the number of the figure selected as the self-body size.

##### Self-concept perception

The Spanish version (40) of the Physical Self-Perception Profile (41) was used to assess physical self-concept. The complete instrument comprises 28 items that assess five factors: fitness (five items, e.g., “I feel very confident about continuously practicing and maintaining my physical form.” α = 0.78; Ω = 0.78); perceived competence (four items, e.g., “I am very good at nearly all sports.” α = 0.78; Ω = 0.79); physical strength (six items, e.g., “I am the first to put myself forward in situations that require strength.” α = 0.68; Ω = 0.65); appearance (nine items, e.g., “I feel very satisfied with how I am physically.” α = 0.72; Ω = 0.74); and self-esteem (four items, e.g., “I don't feel very self-confident in terms of physical appearance” α = 0.65; Ω = 0.65). Responses were rated on a Likert-type scale from 1 (*strongly disagree*) to 4 (*strongly agree*). The instrument evidenced acceptable internal consistency.

##### Physical activity

Physical activity was assessed using the Physical Activity Questionnaire for Adolescents: (PAQ-A) (42). This questionnaire comprises nine items that assess the physical activity pursued by the adolescent over the past 7 days, using a five-point Likert scale: during their free time, during physical education classes and also at different times during class days (lunchtime, afternoons, and evenings), and during the weekend. The result is a score from 1 to 5 that enables the level of physical activity to be graded (43), and their final score is obtained using the arithmetic mean of eight of the nine items, as the last item assesses whether the participant had been ill over the last week (43). Last, the Cronbach's alpha coefficient obtained for this sample was (α = 0.79; Ω = 0.78).

##### Intention to be physically active

This was assessed using an item in which the participant was asked: *Do you intend to practice sport in the future?* The response options were represented by a five-point Likert scale ranging from 1 (*totally disagree*) to 5 (*totally agree*).

### Procedure

Parents and school supervisors were informed by letter about the nature and purpose of the study, and written informed consent was provided. The study obtained permission from the University Ethics Committee, which adheres to the code of ethics established by the World Medical Association (Declaration of Helsinki), and the protocol was approved by the Ethics Committee of the University of Extremadura (89/2016). All participants were treated in accordance with guidelines set out by the American Psychological Association (APA), ensuring trust and anonymity in terms of student responses. Before commencing the data collection process, permission was requested via a letter of consent addressed to parents and teachers, in which the following were to be assessed: What the study consisted of, the anthropometric and psychometric variables that would be assessed and, last, an assurance that all responses would be anonymous and would not compromise the identity of the participants. The participants completed the questionnaires during physical education class in the presence of the teacher, who told them that it was not an assessment test, and that they should be sincere with their answers. While they were completing them, researchers called the students in order from the list to weigh and measure them, subsequently noting down the weight and height in the relevant space on the questionnaire. The results obtained from the tests were passed on to the teacher if they asked for them.

### Statistical Analysis

The SPSS 23.0 statistical package was used to carry out data analysis (see Supplementary Material). Likewise, different tests were used to determine the nature of this data—the Kolmogorov–Smirnov test and Levene's test—which provided scores over 0.05, whereby a decision was made to produce parametric statistics. Descriptive statistics were then provided together with a correlation analysis.

The MPLUS 7.0 statistical package was also used to ascertain predictive capacity (structural equation model) regarding the intention to pursue physical activity, and last, the indirect effects of the structural equations were then calculated.

## Results

Table 1 shows the descriptive statistics and bivariate correlations of all the variables involved in the study, in which significant positive relationships between the intention to pursue physical activity, the time spent on extra-curricular activities, and the dimensions of perceived self-concept are provided. Likewise, it is shown how physical activity is positively related to the perception of self-concept and cardiovascular fitness, and negatively related to agility, anthropometric variables, and the perception of body image. The global score regarding the perception of self-concept also has a significant negative relationship with all those variables that refer to anthropometric values (BMI, waist–height perimeter), body image, and levels of physical condition (agility), except for resistance.

**Table 1:** Descriptive statistics and correlation analysis.

	**1**	**2**	**3**	**4**	**5**	**6**	**7**	**8**	**9**	**10**	**11**	**12**	**13**
1. INPFA	–	0.37^**^	0.17^**^	0.33^**^	0.19^**^	0.11	0.32^**^	0.32^**^	−0.05	0.10	−0.03	−0.061	−0.11^*^
2. PPC	–	–	0.41^**^	0.69^**^	0.43^**^	0.22^**^	0.53^**^	0.75^**^	0.30^**^	0.26^**^	−0.24^**^	0.38^**^	−0.26^**^
3. PAP	–	–	–	0.30^**^	0.44^**^	0.59^**^	0.18^*^	0.73^**^	−0.20^**^	0.11^*^	−0.05	−0.25^**^	−0.17^**^
4. PC	–	–	–	–	0.51^**^	0.21^**^	0.52^**^	0.75^**^	−0.25^**^	0.25^**^	−0.32^**^	−0.28^**^	−0.22^**^
5. PS	–	–	–	–	–	0.34^**^	0.31^**^	0.74^**^	0.04	0.16^**^	−0.13^**^	−0.07	−0.01
6. SEL	–	–	–	–	–	–	0.12^*^	0.66^*^	−0.10	0.06	−03	−0.13	−0.10
7. PA	–	–	–	–	–	–	–	0.46^**^	−0.11^**^	0.21^**^	−0.16^**^	−0.19^**^	−0.12^*^
8. GS_SC	–	–	–	–	–	–	–	–	−0.22^**^	0.23^**^	−0.21^**^	−0.30^**^	−21^**^
9. BMI	–	–	–	–	–	–	–	–	–	−0.17^**^	0.25^**^	0.58^**^	0.73^**^
10. TM6	–	–	–	–	–	–	–	–	–	–	−0.46^**^	−0.13^*^	−0.09
11. Agil	–	–	–	–	–	–	–	–	–	–	–	17^**^	23^**^
12. Body_I	–	–	–	–	–	–	–	–	–	–	–	–	54^**^
13. Wai_hei	–	–	–	–	–	–	–	–	–	–	–	–	–
M	4.6	3.8	3.7	3.5	3.3	3.9	3.2	3.7	18.6	15.8	13.1	3.3	42.4
SD	0.69	0.84	0.70	0.86	0.84	0.96	0.67	0.61	3.00	3.93	1.69	1.13	4.64
Ω	–	0.78	0.72	0.78	0.68	0.65	0.79	–	–	–	–	–	–
Ω	–	0.78	0.74	0.79	0.65	0.65	0.78	–	–	–	–	–	–

*INPFA, intention to pursue physical activity; PPC, perceived physical condition; PAP, perceived appearance; PC, perceived competence; PS, perceived strength; SEL, self-esteem; PA, physical activity; GS_SC, self-concept global score; BMI, body mass index; TM6, 6-min test; Agil, agility; Body_I, body image; Wai_hei, waist–height perimeter*.

* p < 0.01,

** *p < 0.001*.

### Structural Equation Model

A structural equation model was hypothesized with the following structure (Figure 1). This model was formed from three latent variables: physical condition (agility and resistance), anthropometric variables (waist–height perimeter and body mass index), and self-concept (self-esteem, strength, and perceived competence). Variables pertaining to physical activity, extra-curricular time, and intention to be physically active were also used, with the last mentioned acting as a dependent variable of the model.

**Figure 2:**
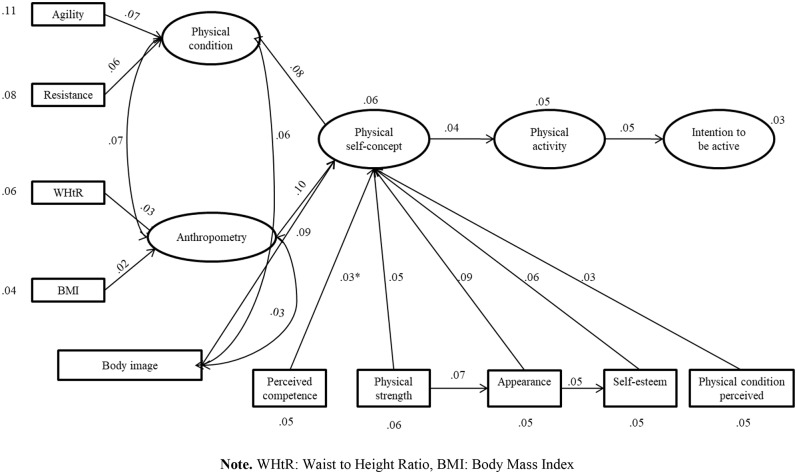
Final model. WHtR, waist-to-height ratio; BMI, body mass index.

The initial model showed the following adjustment indexes—MRLχ^2^ = 372.295, *p* < 0.05, *df* = 66, CFI = 0.74, TLI = 0.68, SRMR = 0.12, and RMSA = 0.12—whereby we decided to restructure the model in accordance with previous literature (9, 22, 24, 30, 33). Thus, the modification indexes suggest new forms of interaction to us: on the one hand, a correlation between body image and anthropometric variables, which provided us with the following adjustment indexes—MRLχ^2^ = 248.954, *p* < 0.05, *df* = 66, CFI = 0.84, TLI = 0.80, SRMR = 0.084, and RMSA = 0.09—and, on the other, a regression in which perceived appearance would predict self-esteem, namely, MRLχ^2^ = 154.238, *p* < 0.05, *df* = 66, CFI = 0.92, TLI = 0.89, SRMR = 0.072, and RMSA = 0.073. This last-mentioned modification provides us with nearly acceptable adjustment indexes, although a decision was made to make a final modification in which perceived appearance would predict strength. The final model (Figure 2) offered some good adjustment indexes: MRLχ^2^ = 125.535, *p* < 0.05, *df* = 66, CFI = 0.94, TLI = 0.92, SRMR = 0.054, and RMSA = 0.059.

Invariance of the model was then tested in terms of gender. To do so, a first step was taken in which it was noted how the model adapted according to participants' gender. The results showed how the model is better explained in the female gender MRLχ^2^ = 64.433, *p* < 0.05, CFI = 0.98, TLI = 0.98, SRMR = 0.046, and RMSA = 0.027, than in the male gender MRLχ^2^ = 131.375, *p* < 0.05, CFI = 0.91, TLI = 0.88, SRMR = 0.072, and RMSA = 0.092. In this respect, the model explains how anthropometric variables, physical condition, and self-concept can be considered greater predictors of the intention to pursue physical activity in the male gender than in the female gender.

Indirect effects among variables were calculated on several levels according to the hypothesized model. The first level showed the indirect effects among all the variables situated between the pursuit of physical activity and physical condition, anthropometric variables, and body image. In this sense, the analysis of indirect effects evidenced significant relationships between physical condition (*p* = 0.001) and PA levels, according to the perception of self-concept [β = 0.231, 95% BcCI = (0.055, 0.212)], in a similar way to how body image showed significant relationships between physical condition (*p* = 0.003) and levels of physical activity, according to the perception of self-concept [β = 0.172, 95% BcCI = (0.058, −0.295)]. However, anthropometric variables proved not to be related to any significant extent (*p* < 0.05) to physical activity, according to the perception of self-concept. The second level covered the indirect effects between the intention to be physically active and self-concept, which showed a significant relationship between the perception of self-concept (*p* = 0.000) and the intention to be physically active, according to physical activity [β = 0.196, 95% BcCI = (0.036, −0.543)]. Last, the third level showed significant relationships between physical condition (*p* = 0.001) and the intention to pursue physical activity, according to the level of that activity [β = 0.074, 95% BcCI = (0.022, −0.362)], in the same way that body image proved to be significantly related (*p* = 0.007) to the intention to be physically active, according to the level of physical activity [β = −0.055, 95% BcCI = (0.020, −0.708)]. For their part, anthropometric variables proved not to be significantly related to the intention to pursue physical activity (*p* < 0.05).

## Discussion

The main purpose of this study was to analyze the relationships established between anthropometric values, body image, physical ability, and the intention to be physically active. A further objective was to test a structural equation model in which physical conditions, anthropometric values, body image, self-concept, and physical activity explain the intention to be physically active.

In terms of the relationships established, a significant positive relationship between the intention to be physically active, physical activity, and the perception of self-concept was shown, and to this end, Zamani Sani et al. (29) showed positive associations between physical activity and self-concept, also drawing attention to the fact that this association is influenced by different key factors such as body image, body mass index, and physical form. In terms of the relationships established between self-concept and the intention to be physically active, the study carried out by Moreno et al. (24) showed significant relationships between the two. Conversely, our study revealed that the intention to pursue physical activity is negatively—albeit not significantly—related to anthropometric values, and to physical form in terms of agility, unlike physical resistance, with which a positive relationship was found. This is in line with previous studies in which attention was drawn to the perception of physical condition as being the most determining factor in being active (44, 45). As for the negative relationship with anthropometric values, our results are in line with those found by Centeio et al. (46), in which physical ability acted as a mediator with the intention to be physically active, although the intention to pursue physical activity may vary depending on the strategies we implement. In this respect, García-Hermoso et al. (47) point out that the intention to be physically active might be improved by activities such as walking or cycling to school. Likewise, the overall perception of self-concept was negatively and significantly related to anthropometric values, and agility to physical form, although this proved not to be the case with resistance. In this sense, Reigal-Garrido et al. (48) draw attention to the fact that the study of aerobic capacity is a determining factor in the perception of self-concept.

In terms of the structural equation model, our model showed that physical condition, anthropometric variables, and body image predict the perception of physical self-concept as backed up by previous literature on the subject. As for the predictive role of physical condition, Garn et al. (23) draw attention to the fact that overall physical self-concept is predicted by multiple improvements that take place in physical condition. Thus, the fact might be interpreted that improvements taking place via specific exercises involving speed, strength, and resistance would have a direct influence on self-concept, as shown by Lemoyne et al. (49) in university students. On the other hand, and in terms of the predictive role of anthropometric values (BMI and waist–height perimeter), our results are in keeping with those found in the study by Fernández-Bustos et al. (9), where a model was shown in which BMI predicted physical activity and self-concept. A difference should also be highlighted in the role taken on by body image in the model put forward by Fernández-Bustos et al. (9) and ours. In this respect, they provided body image with a mediating role between BMI and physical activity (50), whereas our study showed body image to be an independent construct which, alongside anthropometric values and variables associated with physical ability, predict self-concept. Therefore, it should be noted that anthropometric variables such as BMI are associated with body dissatisfaction in both men and women (10).

Last, our model showed the role played by self-concept in pursuing or intending to pursue physical activity. These findings are in keeping with those made by Moreno et al. (24), in which it was shown that physical self-concept predicted the intention to pursue physical activity, although the intention that a subject should remain physically active is influenced by the perception of physical self-concept, as this is positively modified via physical activity (51), and also with the findings made by Fernandez-Rio et al. (31), in which the pursuit of physical activity predicted intention. To this, we should also add the importance of other variables such as motivation and enjoyment that will influence the intention to pursue such activity (52).

Nonetheless, the results obtained should be treated with caution, as the present research is subject to certain limitations such as its transversal design, which does not enable cause–effect relationships to be established, and the small number of participants, whereby these conclusions are not able to be transferred to a different population. Thus, this research draws the conclusion that the intention to pursue and practice physical activity is predicted by body image, anthropometric values, physical condition, and the perception of physical self-concept. In this respect, those individuals who evidence having a healthy body image accompanied by suitable anthropometric values and physical condition will be more likely to have a perception of self-concept and, therefore, be more inclined to pursue physical activity. Future studies should replicate this same hypothesized model, increasing the sample size. On another hand, it should focus on stipulating profiles of physical self-concept and being more specific about these relationships, rather than referring to a high or low degree of self-concept, as this might give rise to incongruences when establishing such relationships. In this regard, there may, on the one hand, be individuals who have a great perception of strength and physical competence, who maintain more significant relationships with tests associated with physical ability, while on the other, other types of individual may have a better self-esteem and perception of themselves and be more closely related to variables such as body image.

## Data Availability Statement

All datasets generated for this study are included in the article/Supplementary Material.

## Ethics Statement

This study was conducted in accordance with the Declaration of Helsinki, and the protocol was approved by the Ethics Committee of the University of Extremadura (89/2016). Written informed consent to participate in this study was provided by the participants' legal guardian/next of kin.

## Author Contributions

PS-M, PL-G, ML-G, and MT-S contributed conception and design of the study. DH-A and MV-S organized the database. PS-M and MV-S performed the statistical analysis and wrote the first draft of the manuscript. PS-M, MT-S, and MV-S wrote sections of the manuscript. All authors contributed to manuscript revision, read, and approved the submitted version.

## Conflict of Interest

The authors declare that the research was conducted in the absence of any commercial or financial relationships that could be construed as a potential conflict of interest.
